# The Silk-protein Sericin Induces Rapid Melanization of Cultured Primary Human Retinal Pigment Epithelial Cells by Activating the NF-κB Pathway

**DOI:** 10.1038/srep22671

**Published:** 2016-03-04

**Authors:** J. R. Eidet, S. Reppe, L. Pasovic, O. K. Olstad, T. Lyberg, A. Z. Khan, I. G. Fostad, D. F. Chen, T. P. Utheim

**Affiliations:** 1 Department of Ophthalmology, Oslo University Hospital, Oslo, Norway; 2 Department of Medical Biochemistry, Oslo University Hospital, Oslo, Norway; 3 Faculty of Medicine, University of Oslo, Oslo, Norway; 4 Department of Oral Biology, Faculty of Dentistry, University of Oslo, Oslo, Norway; 5 Schepens Eye Research Institute, Harvard Medical School/Massachusetts Eye and Ear, Boston, MA.

## Abstract

Restoration of the retinal pigment epithelial (RPE) cells to prevent further loss of vision in patients with age-related macular degeneration represents a promising novel treatment modality. Development of RPE transplants, however, requires up to 3 months of cell differentiation. We explored whether the silk protein sericin can induce maturation of primary human retinal pigment epithelial (hRPE) cells. Microarray analysis demonstrated that sericin up-regulated RPE-associated transcripts (RPE65 and CRALBP). Upstream analysis identified the NF-κB pathway as one of the top sericin-induced regulators. ELISA confirmed that sericin stimulates the main NF-κB pathway. Increased levels of RPE-associated proteins (RPE65 and the pigment melanin) in the sericin-supplemented cultures were confirmed by western blot, spectrophotometry and transmission electron microscopy. Sericin also increased cell density and reduced cell death following serum starvation in culture. Inclusion of NF-κB agonists and antagonists in the culture medium showed that activation of the NF-κB pathway appears to be necessary, but not sufficient, for sericin-induced RPE pigmentation. We conclude that sericin promotes pigmentation of cultured primary hRPE cells by activating the main NF-κB pathway. Sericin’s potential role in culture protocols for rapid differentiation of hRPE cells derived from embryonic or induced pluripotent stem cells should be investigated.

Viable retinal pigment epithelial (RPE) cells are critical for normal photoreceptor cell metabolism and visual cycle. Hence, RPE degeneration is accompanied by concomitant photoreceptor degeneration^1^. Drusen, which are located between the RPE cells and Bruch’s membranes, contain a number of proteins that relate to inflammation; and there is increasing body of evidence that oxidative stress^2^, local inflammation^3^, and activation of the complement system^4^ play a part in the development of AMD. The choriocapillaris has also been suggested to play a role in the etiology of AMD^5^. The disorder is subdivided into a dry and a wet type. There are no established curative treatment options for the dry type of AMD, which constitutes more than 85% of the cases. Cell based restoration of RPE cells has been explored in several studies^6^,^7^. There are two main approaches for delivering cultured RPE to the submacular space: 1) injection of a RPE cell suspension; and 2) implantation of RPE as an intact cell sheet^8^. Benefits of the former include ease of procedure, while some of the disadvantages are increased apoptosis due to loss of cell-cell interaction and a disorganized RPE upon injection^8^. Implantation of RPE as an intact cell sheet should in theory prevent the mentioned disadvantages of injecting RPE.

Several culture protocols intended for driving the differentiation of human embryonic stem cells (hESC) and human induced pluripotent stem cells (iPSC) into RPE have been described^9^,^10^,^11^,^12^. Although protocols using hESC or iPSC have successfully produced differentiated and pigmented RPE cells, they are usually time-consuming, sometimes necessitating a total culture period of up to 3 months^13^. Cutting down the time necessary for producing differentiated RPE reduces costs of production and lessens the risk of infection during prolonged cultures.

The silk protein sericin mainly consist of three polypeptides having molecular masses of 400, 250, and 150 kDa^14^ and encompasses 25–30% of all silk proteins^15^. It has been recognized to increase cell proliferation in serum-free media^16^, promote antioxidant effects^17^, inhibit the melanogenesis enzyme tyrosinase^17^, enhance wound healing^18^ and support the growth and attachment of fibroblasts^18^.

Sericin has demonstrated serum-sparing effects in media for cryopreservation of human dermal fibroblasts, human epidermal keratinocytes and rat islets^19^,^20^. The prospect of avoiding serum, either autologous or animal-derived, has become increasingly appealing with steadily tougher regulatory demands in regenerative medicine over the past years. Replacing serum with sericin could dramatically reduce the risk of infections, which for animal derived components may be particularly severe, not only for the patient, but also for the larger community^21^. Despite the rare occurrence of infections following the use of fetal bovine serum (FBS), measures taken to avoid both the risk of infections and standardize the culture protocols are most welcomed by regulatory authorities. In the current study, we explored the potential of using the silk protein sericin as serum-substitute in culture medium to induce maturation of primary human retinal pigment epithelial cells (hRPE). Our results revealed a surprising effect of sericin on promoting hRPE maturation by activating the NF-κB pathway.

## Results

### Microarray Analysis of hRPE Cultured With or Without Sericin

Microarray experiments were performed to assess the effect on hRPE of adding sericin to the culture medium in absence of FBS. Similarly as described in materials and methods, hRPE were seeded in complete EpiCM on Nunclon Δ surface plates and cultured for two days before replacing EpiCM with either 1) DMEM with 1% sericin or 2) DMEM. Both DMEM media were supplied with 10.000 U penicillin and 10 mg streptomycin at a final concentration of 1%.

#### Global Perspective

Gene expression differed considerably between the two culture groups, with a total of 438 significantly differentially regulated genes (fold change >1.5; *P* < 0.05). In the sericin-supplemented group, 229 genes were down-regulated and 209 genes were up-regulated compared to the control, while 14419 transcripts were unchanged.

#### Upstream analysis

**Figure 1:**
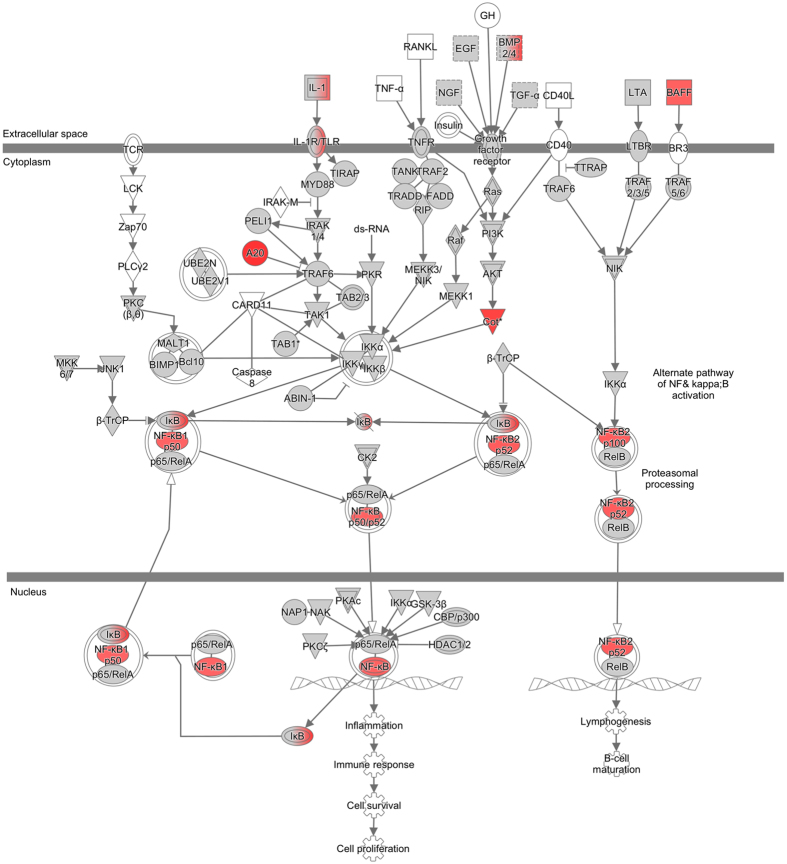
Pathway analysis. Transcripts that changed upon addition of sericin were assessed by Ingenuity Pathway Analysis (IPA) to discover enriched functional pathways. The NF-κB complex was among the most highly activated (Z-score: 4.597) upstream regulators. IPA revealed up-regulation (red symbols) of transcripts belonging to both the main and alternate NF-κB pathway.

The top upstream regulators were tumor necrosis factor (TNF) (z-score: 5.008), interferon-γ (IFN-γ) (z-score: 4.623), the NF-κB complex (z-score: 4.597) and interleukin-1ß (IL1ß) (z-score: 4.567). Pathway analysis of NF-κB revealed up-regulation of transcripts belonging to both the main and alternate NF-κB pathway (Fig. 1). Activation of the NF-κB pathway was indicated by up-regulation of several of its member transcripts, including *TNFAIP3, MAP3K8* and *NFKB2* (Table 1).

#### Substantially Regulated Genes

The C-X-C motif chemokine 10 (*CXCL10*, also known as IP-10; interferon gamma-induced protein 10) was the most up-regulated gene in the dataset, with a 55.5-fold up-regulation in the sericin-supplemented group compared to the control (Table 2). Complement component 3 (*C3*) was the second most up-regulated gene in the sericin group, with a 34.7-fold up-regulation. The chemokine (C-C motif) ligand 2 (*CCL2*) was up-regulated 21.6-fold in the sericin-supplemented group (Table 2). *TNFAIP3* (tumor necrosis factor, alpha-induced protein 3; *A20*), which is involved in NF-κB regulation, was up-regulated 10.4-fold in the sericin group.

#### Downstream Effects

**Figure 2:**
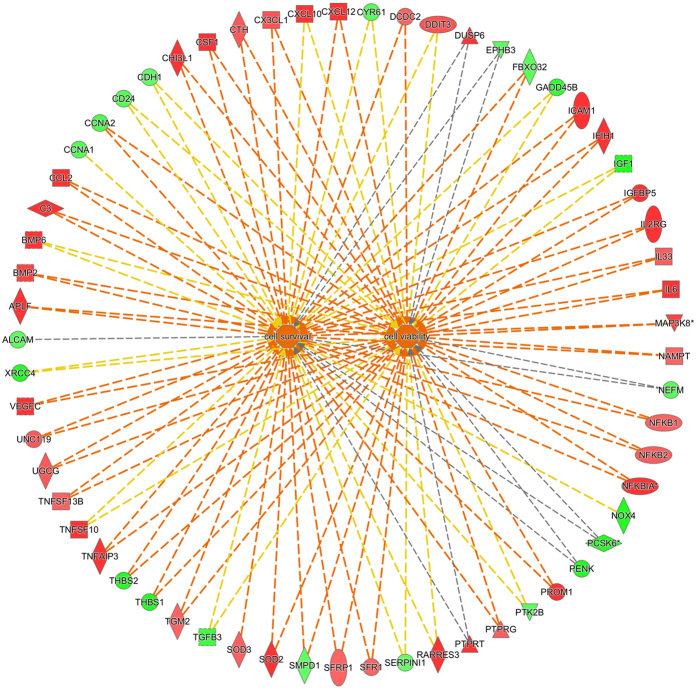
Downstream effect upon culturing of human retinal pigment epithelial cells with or without sericin. Ingenuity Pathway Analysis predicted a downstream effect compatible with increased viability and survival of human retinal pigment epithelial cells cultured in DMEM with 1% sericin compared to cells cultured without sericin. In the cell viability category (right center), 37 of 57 genes had an expression direction consistent with increased cell viability, yielding a Z-score of 2.8. In the cell survival category (left center), 38 of 61 genes had an expression direction consistent with increased cell survival, yielding a Z-score of 2.7. Red symbols indicate increased transcript levels upon culturing in presence of sericin, while green symbol indicate reduced transcript levels. The dotted lines indicate indirect relationships leading to activation (orange) or inhibition (blue). Yellow and grey dotted lines indicate inconsistent relationships and no predicted effects, respectively.

The results obtained from the Ingenuity Pathway Analysis (IPA) predicted a downstream effect compatible with increased cell viability and survival in the sericin-supplemented group (Fig. 2). In the cell viability category, 37 of 57 genes had an expression direction consistent with increased cell viability, yielding a Z-score of 2.8. In the cell survival category, 38 of 61 genes had an expression direction consistent with increased cell survival, yielding a Z-score of 2.7.

#### RPE Related Gene Transcripts

##### Pigmentation

The (IPA) detected 11 differentially expressed genes related to cell pigmentation (Table 3). Ten of the genes were up-regulated and one was down-regulated in hRPE cells cultured in DMEM with sericin compared to cells cultured in DMEM without sericin.

#### Visual Cycle

Several visual cycle genes were significantly up-regulated in hRPE cells cultured in DMEM with sericin compared to that of cells cultured in DMEM without sericin, while no visual cycle genes were significantly down-regulated (Table 4). Compared to cells cultured in DMEM without sericin, the cells that were cultured in DMEM with sericin displayed a 2.8-fold up-regulation of *RPE65*, a key isomerase of the visual cycle and an essential marker of RPE cell maturation^22^,^23^, and a 1.4-fold up-regulation of cellular retinaldehyde binding protein 1 (*RLBP1*; also known as *CRALBP*), which positions retinol for enzymatic turnover and thereby accelerates the process^24^. Both retinol dehydrogenase 10 (RDH10) and retinol dehydrogenase 11 (RDH11) play complementary roles as 11-cis-retinol dehydrogenases in the visual cycle^25^,^26^,^27^, and were up-regulated 1.9 and 1.6-fold, respectively, in cells cultured in DMEM with sericin compared to cells cultured in DMEM without sericin.

### RT-PCR Validation

To validate the microarray results, *NFKBIA, RPE65*, CSF1, *NFKBIZ* and *RGR* transcripts were quantified by RT-PCR in hRPE cells cultured in DMEM with or without 1% sericin for a total of 14 days. In Table 5, ΔΔCt values are transformed to fold change. All six transcripts were up-regulated in the Affymetrix and RT-PCR experiments (Table 5).

### Microstructure Following hRPE Culture With or Without Sericin

**Figure 3:**
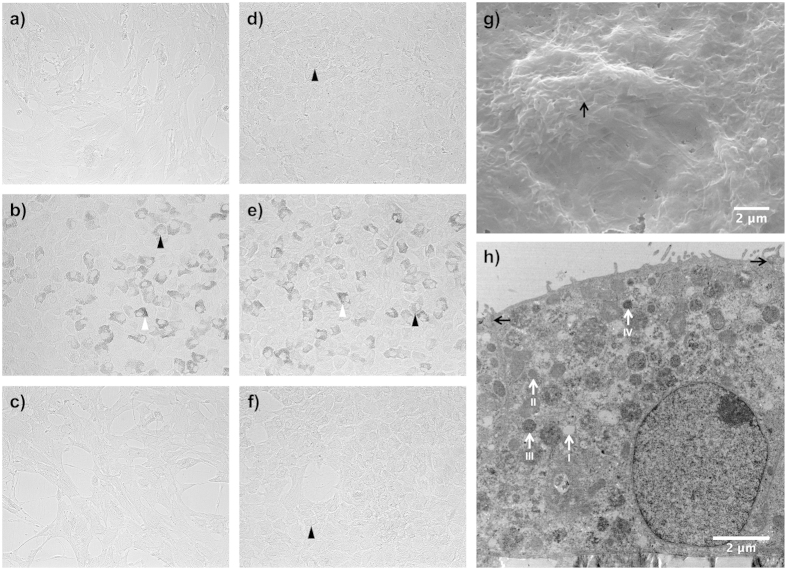
Micro- and ultrastructure of human retinal pigment epithelial cells cultured with or without sericin. Photomicrographs (**a–f**) show human retinal pigment epithelial cells cultured for 12 days with or without 1% sericin or 10% fetal bovine serum (FBS) using two different basal media. (**a**) DMEM/FBS; (**b**) DMEM/sericin; (**c**) DMEM; (**d**) MEM-α/FBS; (**e**) MEM-α/sericin; (**f**) MEM-α. Cellular pigmentation is indicated by white arrowheads and cobblestone morphology is indicated with black arrowheads. Magnification: 200×. Photomicrographs were captured by a black-and-white camera and are representative of eight samples. Electron microscopy analyses (**g,h**) were performed on human retinal pigment epithelial (hRPE) cells cultured in DMEM with 1% sericin for 12 days. (**g**) Scanning electron microscopy image showing the apical surface of a confluent layer of hexagonal hRPE cells with extensive microvilli (arrow). Image is representative of three samples. (**h**) Transmission electron microscopy image showing a polarized hRPE cell with apical tight junctions (black arrows), a basal nuclei and numerous melanosomes between stage I (non-melanized) and stage IV (melanized). Image is representative of four samples.

Human retinal pigment epithelial cells were cultured with or without 1% sericin in two different basal media with or without FBS for 12 days before being assessed with light microscope for morphology and pigmentation. Widespread pigmentation was only seen in cells cultured with 1% sericin (DMEM/sericin; Fig. 3b, MEM-α/sericin; Fig. 3e), irrespective of the basal medium used (MEM-α or DMEM). Pigmented cells were rarely found in the groups that were cultivated without sericin (DMEM/FBS; Fig. 3a, DMEM; Fig. 3c, MEM-α/FBS; Fig. 3d, MEM-α; Fig. 3f). Typical cobblestone morphology with essentially hexagonal cells was seen in the samples cultured in DMEM with sericin (Fig. 3b), MEM-α with sericin (Fig. 3e), MEM-α with FBS (Fig. 3d), and MEM-α alone (Fig. 3f). Cell confluence was superior in DMEM with 1% sericin (Fig. 3b) compared to DMEM alone (Fig. 3c) and DMEM with 1% FBS (Fig. 3a). Hence, these results show that after 12 days in culture, only cells cultured in media supplied with sericin become pigmented and sericin appears to support hRPE cell confluence.

### Ultrastructure Following hRPE Culture With or Without Sericin

Scanning electron microscopy of hRPE cultured for 12 days in DMEM with 1% sericin appeared tightly adjoined, hexagonal and had apical microvilli (Fig. 3g), hence demonstrating a differentiated ultrastructure. Transmission electron microscopy of hRPE cultured for 12 days in DMEM with 1% sericin contained melanosomes of all four stages following seven days of culture (Fig. 3h), thereby supporting the validity of the light microscopy experiments.

### Melanin Quantification Following hRPE Culture With or Without Sericin

**Figure 4:**
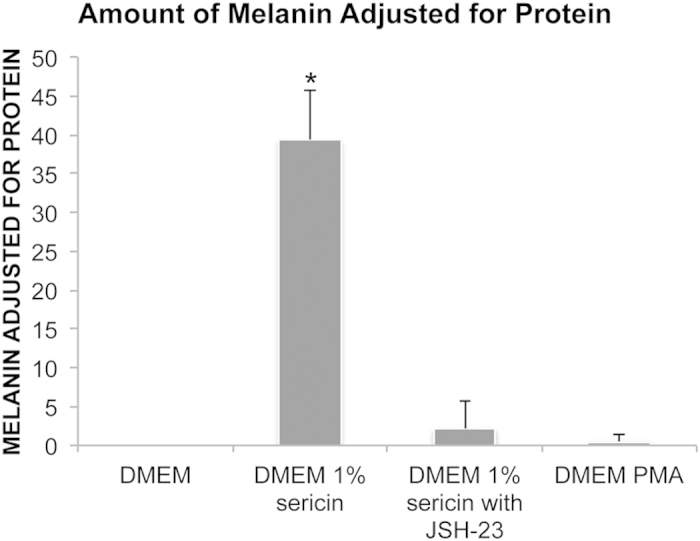
Effect of sericin on melanin production. The bar chart shows levels of melanin adjusted for total protein after culturing human retinal pigment epithelial cells for seven days in DMEM with or without 1% sericin, the NF-κB activator inhibitor 4-methyl-N1-(3-phenylpropyl)-1,2-benzenediamine (JSH-23) and the NF-κB agonist phorbol-12-myristate-13-acetate (PMA). N = 3. Error bars: standard deviation. *P < 0.05 compared to all other groups.

As the pigment melanin absorbs light at a specific wave length, measurement of pigment quantity is commonly performed by spectrophotometry^28^. Following 12-day culture, spectrophotometry showed increased absorption at 562 nm in cells cultured in DMEM with 1% sericin (39 ± 6 melanin/protein; *P < *0.001) compared to cells cultured in DMEM without sericin (Fig. 4). Thus, the results further supports increased pigmentation in sericin-cultured cells.

### Protein Levels of CRALBP and RPE65 Following hRPE Culture With or Without Sericin

**Figure 5:**
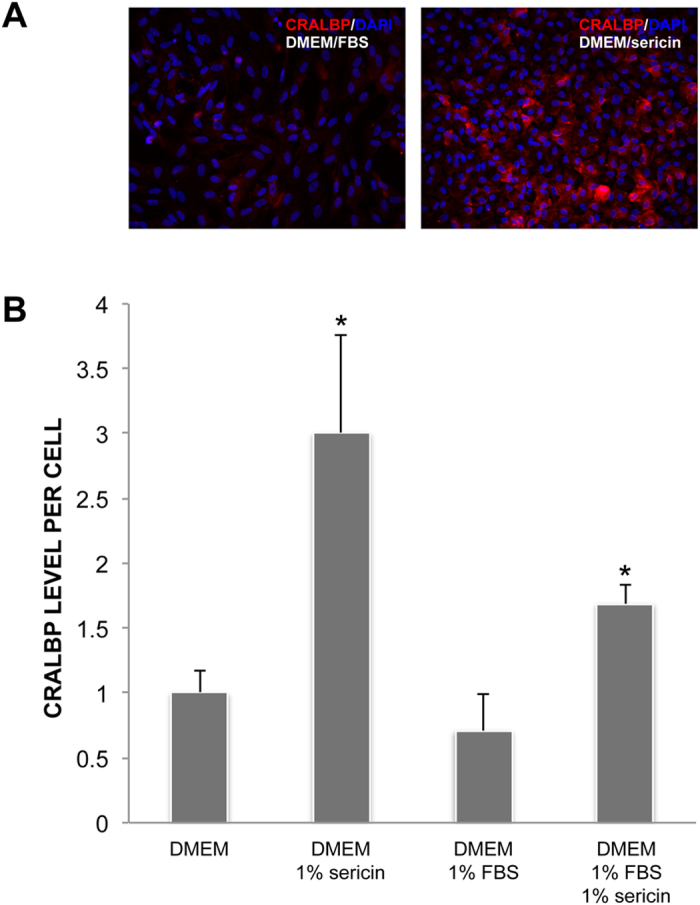
Assessment of CRALBP levels by immunofluorescence. The expression of the cellular retinaldehyde-binding protein (CRALBP) was measured by quantitative immunofluorescence in human retinal pigment epithelial cells cultured for 12 days in DMEM with or without 1% sericin and/or fetal bovine serum (FBS). (**a**) Photomicrographs show that CRALBP expression (red) was lowest in the DMEM/FBS group and highest in the DMEM/sericin group. Cell nuclei were counterstained with DAPI (blue). Magnification: 200×. Photomicrographs are representative of four to eight samples. (**b**) Bar chart showing CRALBP expression in fold change relative to the DMEM group. N = 4 to 8. Error bars: standard deviation. *P < 0.01 compared to all other groups.

To verify the presence and to assess the quantity of RPE-related proteins quantitative immunofluorescence and western blot experiments were used to analyze the mean levels of CRALBP and RPE65. With quantitative immunofluorescence, CRALBP was most abundant in cells cultured for 14 days in DMEM with sericin, and present to a significantly higher degree than in cells cultured in DMEM alone, DMEM with FBS or DMEM with FBS and sericin (*P* < 0.01) (Fig. 5). Retinal pigment epithelial cells cultured in DMEM with sericin and FBS also demonstrated a significantly higher expression of CRALBP compared to the DMEM alone and DMEM with FBS groups (*P* < 0.01). Thus, quantitative immunofluorescence confirmed increased levels of CRALBP, as indicated by the microarray results (Table 4). On western blot experiments, however, the level of CRALBP protein in cells cultured in DMEM with sericin (87% ± 21%) relative to that in cells cultured in DMEM alone (100%; *P* = 0.38) was similar. The level of RPE65 protein, on the other hand, was significantly higher in the DMEM with sericin group (168% ± 11%) compared to in DMEM alone (100%; *P* = 0.008), as measured by western blot. Therefore, sericin increases RPE65 protein in cultured primary hRPE.

### hRPE Cell Density and Cell Death Following Serum Starvation

**Figure 6:**
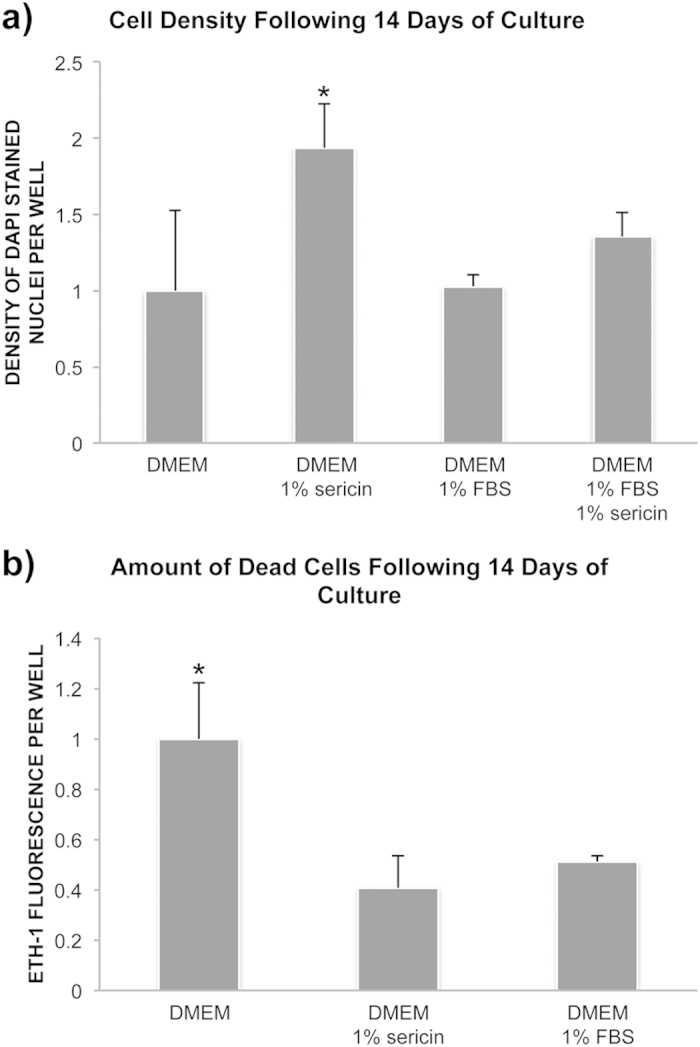
Effect of sericin on cell survival. To assess the effect of sericin on cell survival confluent layers of human retinal pigment epithelial cells were cultured in DMEM with or without sericin or fetal bovine serum (FBS) for 12 days. (**a**) Bar chart showing cell density in fold change relative to the DMEM-group. Cell density was measured by counting DAPI-stained nuclei using ImageJ. N = 4 to 8. Error bars: standard deviation. *P < 0.01 compared to all other groups. (**b**) Bar chart showing amount of dead cells in fold change relative to the DMEM-group. Cell death was measure by ethidium homodimer-1 (ETH-1) uptake by dead cells using a microplate reader. N = 8. Error bars: standard deviation. *P < 0.001 compared to the other two groups.

As the microarray results predicted increased viability of culturing cells in DMEM with sericin compared to DMEM without sericin, cell density and cell death after 12 days of cultivation with or without sericin was quantified by counting DAPI-stained cell nuclei and by microplate fluorometer measurements of EH-1, the latter which is indicative of dead cells. DMEM with sericin yielded the highest cell density and significantly higher density than DMEM alone (*P* = 0.006), DMEM with FBS (*P* < 0.001) and DMEM with sericin and FBS (*P* = 0.007) (Fig. 6a).

Cultured hRPE were assayed with EH-1 to quantify the number of dead cells following 14 days of culture. The highest number of dead cells was obtained when using DMEM alone (Fig. 6b). Cells cultured in this medium demonstrated a significantly higher amount of dead cells than when using DMEM with sericin or DMEM with FBS (*P* < 0.001). Thus, the cell density and cell death results supported the microarray prediction of increased viability when culturing hRPE in DMEM with sericin.

### NF-κB Pathway and Melanization of Cultured hRPE Cells

**Figure 7:**
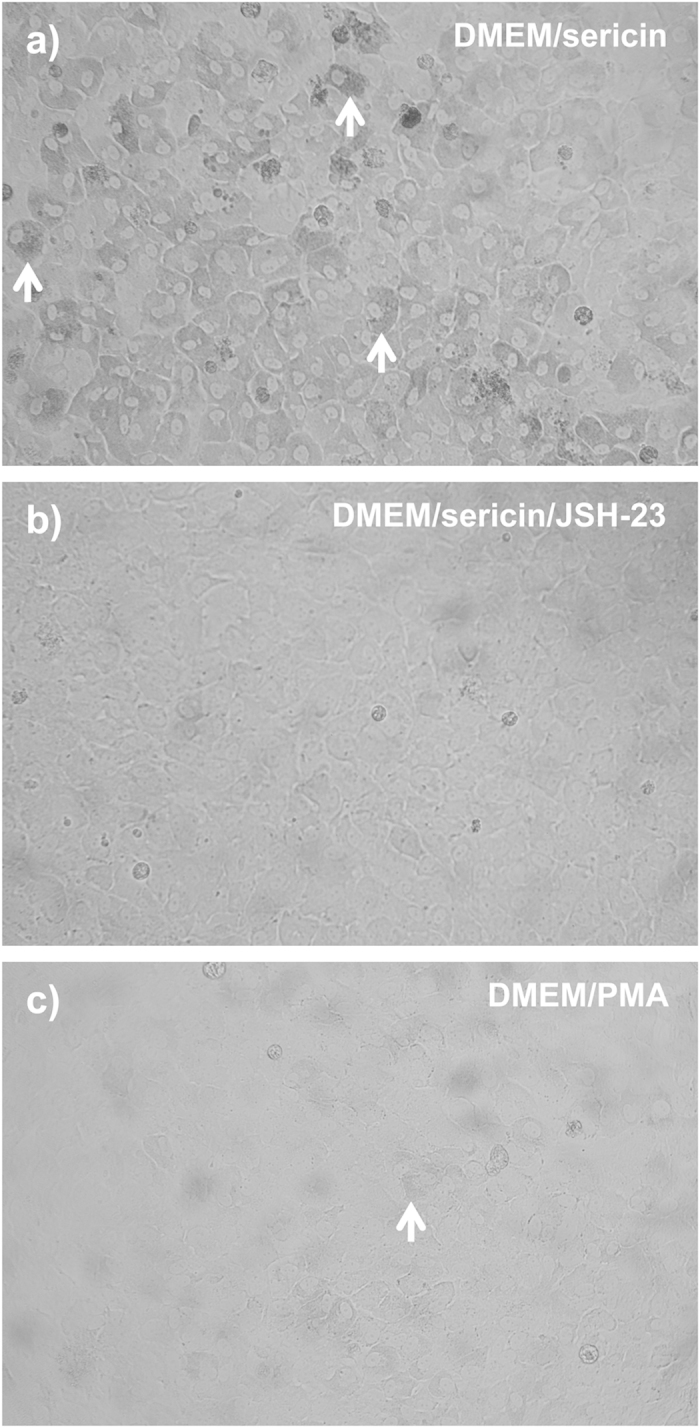
Effect of stimulation/inhibition of NF-κB signaling. The NF-κB activator inhibitor 4-methyl-N1-(3-phenylpropyl)-1,2-benzenediamine (JSH-23) and the NF-κB agonist phorbol-12-myristate-13-acetate (PMA) was used to assess whether activation of the NF-κB pathway is necessary and sufficient for sericin-induced pigmentation of human retinal pigment epithelial (hRPE) cells following seven days of culture. (**a**) Photomicrograph showing pigment-containing hRPE cells (white arrows) following culture in DMEM with sericin. Magnification: 200×. (**b**) Photomicrograph showing absence of pigment in hRPE cells following culture in DMEM with sericin and JSH-23. Magnification: 200×. (**c**) Photomicrograph showing scarce pigment in hRPE cells (white arrow) following culture in DMEM with PMA. Magnification: 200×. Photomicrographs have been converted to gray scale images and are representative of three samples.

Since IPA identified both the NF-κB complex, and TNF, a well known activator of NF-κB^29^, as top upstream regulators, we focused on the role of NF-κB signaling in melanization. The NF-κB-inhibitor JSH-23 specifically inhibits nuclear translocation and activation of NF-κB^30^. The addition of JSH-23 to a culture medium consisting of DMEM with 1% sericin prevented development of pigmented cells as demonstrated by light microscopy and spectrophotometry following seven days of culture, as opposed to control cells cultured in DMEM with sericin, but without JSH-23 (Figs 4 and 7a,b) (N = 3). However, stimulation of the NF-κB pathway by adding PMA, a NF-κB agonist, to a culture medium consisting of DMEM without sericin did not induce melanization (Figs 4 and 7c). Thus, NF-κB activation appears to be necessary, but not sufficient, for sericin-induced melanization of hRPE.

### Effect of Sericin on the Main and Alternate NF-κB Pathways

To verify the microarray data, which indicated that sericin induces activation of the NF-κB pathway, we next investigated sericin’s effect on activation of the main (phospho-NF-κB p65) and alternate (NF-κB p52) pathways. Following 12 days, primary hRPE cells cultured in DMEM with 1% sericin exhibited a higher level of phospho-NF-κB p65 divided by total protein (7.6 ± 0.7) compared to cells cultured in DMEM alone (5.9 ± 0.1; *P* = 0.01). To investigate if FBS inhibits sericin’s stimulatory effect on phospho-NF-κB p65, we next compared cells cultured in DMEM with 1% sericin with that of cells cultured in DMEM with 1% sericin and FBS. The addition of FBS caused an even higher level of phospho-NF-κB p65 (12.4 ± 1.7; *P* = 0.01) compared to the DMEM with 1% sericin group.

**Figure 8:**
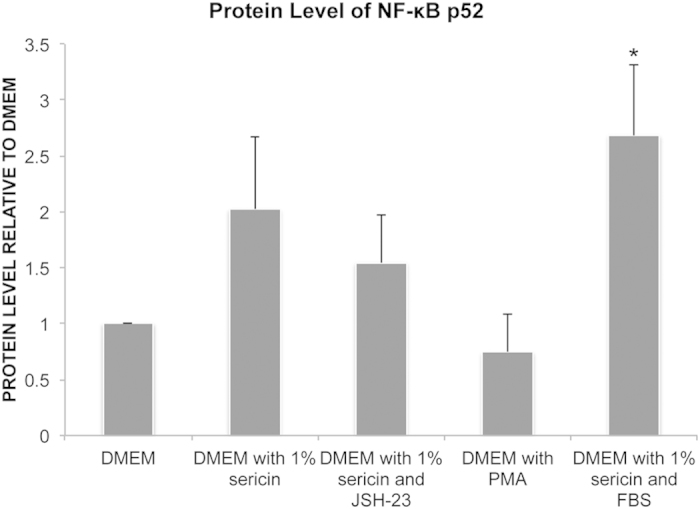
Effect of Sericin on the alternate NF-κB pathway. The effects of sericin, the NF-κB activator inhibitor 4-methyl-N1-(3-phenylpropyl)-1,2-benzenediamine (JSH-23), the NF-κB agonist phorbol-12-myristate-13-acetate (PMA) and fetal bovine serum (FBS) on the protein level of NF-κB p52 in the alternate NF-κB pathway was assessed by western blot on primary human retinal pigment epithelial (hRPE) cells cultured for 12 days. Bar chart shows amount of protein relative to the DMEM group. N = 3. *P = 0.04 compared to DMEM alone.

Regarding the alternate pathway, the mean level of NF-κB p52 protein in cells grown in DMEM with 1% sericin (2.0 ± 0.6; *P* = 0.12) relative to that in cells grown in DMEM alone was approximately two-fold higher, but the potential difference between the groups did not reach significance (N = 3) (Fig. 8). Cells cultured in DMEM with 1% sericin and FBS (2.7 ± 0.6; *P* = 0.04) demonstrated a more than two-fold increase in the level of NF-κB p52 protein relative to cells grown in DMEM alone (Fig. 8). Collectively, these data verify that sericin activates the main NF-κB pathway and that FBS do not inhibit sericin’s activation.

## Discussion

In the current study, sericin induced pigmentation of cultured hRPE by NF-κB pathway activation while still preserving cell viability and improving RPE maturation, as indicated by pathway analysis of Affymetrix microarray, micro and ultra-structural studies, spectrophotometry, western blot, ELISA, and viability assays.

The NF-κB pathway was, alongside TNF, IFN-γ and IL1ß, one of the top upstream sericin-induced regulators in this study. The NF-κB pathway is involved in multiple cellular processes, including inflammation and immunity. NF-κB is also part of the TNF pathway and is regulated by IL1ß. A link between inflammatory cytokines, including IL1ß and TNF-α, and induction of pigmentation in chick RPE cells has been reported^31^. Interestingly, IFN-γ activation has been related to hypopigmentation in skin melanocytes^32^. In our study, sericin activated the main NF-κB pathway. Furthermore, the addition of the NF-κB activator inhibitor JSH-23 prevented pigmentation in sericin-cultured cells and thereby confirmed the role of NF-κB in pigmentation of hRPE, whereas the relatively scarce pigmentation achieved with the NF-κB agonist PMA alone suggested that sericin promotes pigmentation, albeit not exclusively, by activating the NF-κB pathway.

Ingenuity pathway analysis identified several genes that are potentially linked to sericin-induced pigmentation, including *PROM1, C10orf11, SLC24A5, TGM2* and *IRF1*, which were all up-regulated by sericin. *PROM1* is also related to maculopathy, as mutations in this gene may cause macular degeneration, including dominant bull’s eye maculopathy^33^,^34^. Mutations in *C10orf11* have been shown to decrease pigmentation of melanocytes and lead to human albinism^35^. Interestingly, down-regulation of *SLC24A5* causes reduced melanin content in chick RPE^36^. Furthermore, *SLC24A5* has also been related to melanin content in skin melanocytes and the gene product of *SLC24A5* localizes to intracellular membranes, including melanosomes^37^. Inhibition of *TGM2* has been reported to suppress melanogenesis in human melanoma cells^38^. Surprisingly, up-regulation of *IRF1* has been associated with hypo-pigmentation in skin melanocytes^39^.

Previous reports have demonstrated that sericin inhibits tyrosinase, which is the main rate-limiting melanogenesis enzyme^17^, thus the induction of pigmentation by sericin in our study is unexpected. Tyrosinase catalyses the formation of dihydroxyphenylalanine (L-DOPA) from L-tyrosine^40^. L-DOPA is subsequently converted to melanin, aided by tyrosinase-related proteins 1 and 2 (TRP-1 and TRP-2)^41^. Tyrosinase is inhibited by acidic conditions, including those resulting from high metabolic activity^42^. The microarray data did not reveal any significant effect of sericin on the tyrosinase transcript *TYR*, or on TRP-1 and TRP-2 encoding genes, (*TYRP1* and *TYRP2*, respectively), thereby suggesting that sericin’s effect on pigmentation is unrelated to regulation of these genes. The production of melanin is a complex process, however, involving several steps where sericin could have a stimulating role that fully compensates for its acclaimed tyrosinase-inhibiting effect.

Pigmentation was almost exclusively seen in cells that had been cultured in sericin-supplemented basal media (either MEM-α or DMEM). Hexagonal cobblestone morphology was also achieved when using either MEM-α or DMEM supplemented with sericin. After culturing the cell line ARPE-19 for 98 days in DMEM with FBS, Ahmado and co-workers demonstrated pigmented and hexagonal cells^43^. To our knowledge, this medium has not been used for normal RPE. The MEM-α-based medium, however, has been shown to induce pigmentation in normal hRPE cells in study by Sonoda *et al*.^44^. In contrast to our study, the hRPE in their study was pigmented upon start of culture and, after initial depigmentation, became repigmented after 14 days. Both MEM-α and DMEM supplemented with FBS are known to induce maturation of RPE cells in prolonged culture (>3 weeks^44^/months^43^). Our culture time with sericin of seven to 14 days may, therefore, have been too short time for widespread melanogenesis to occur in these media.

In RPE, pigment can be seen within vesicles called melanosomes, which can be divided into four stages of development based on ultrastructure^45^. While stage I and II melanosomes are amelanotic, stage III and IV melanosomes are partially, and fully, pigmented, respectively. hRPE cultured in DMEM containing sericin displayed melanosomes of all four stages following 14 days of culture, which suggests that the process of melanogenesis was ongoing.

The tight junction barrier is involved in creating a polarized epithelium and is necessary for maintaining an apical-basal concentration gradient across the RPE^44^. Thus, as shown by TEM in the current study, sericin enabled the development of a polarized RPE, as indicated by presence of apical tight junctions and basal cell nuclei.

*TNFAIP3, CXCL10, C3* and *CCL2* were among the top five sericin-induced genes in this study. *TNFAIP3* was also the top up-regulated gene in the NF-κB pathway. It is anti-inflammatory and prevents NF-κB and TNF-mediated apoptosis^46^. *TNFAIP3* is induced by TNF and inhibits the NF-κB pathway by de-ubiquitination and ubiquitination of the TNF receptor-interacting protein^47^. Zinc supplementation in human AMD patients, which up-regulates *TNFAIP3*^48^, has been shown to be associated with decreased risk of developing advanced AMD or neo-vascular AMD during a 10-year follow-up^49^. In rats, *TNFAIP3* (*A20*) was identified as a candidate gene for development of retinopathy^50^. The CXCL10 protein is a potent inhibitor of angiogenesis^51^ and an antitumor agent causing tumor necrosis^52^. C3 is commonly found in drusen of AMD patients^53^, and the presence of C3 is critical for protection of the retina^54^,^55^. Absence of *C3* expression has deleterious effects on the retinal structure and leads to progressive retinal degeneration^54^,^55^. C3 can also initiate angiogenesis, thereby opposing the anti-angiogenic effect of CXCL10. CCL2 contributes in maintaining normal RPE morphology, and lack of the gene leads to RPE cell loss and stress^56^.

Inclusion of sericin in the culture medium leads to up-regulation of several genes related to the visual cycle, including *RPE65, RDH10* and *CRALBP*. Retinal diseases can result from mutations or malfunction of key proteins in the visual cycle, in which the RPE serves as a critical component^24^. *The RDH10* is essential for synthesis of embryonic retinoic acid and therefore for limb, craniofacial and organ development^57^. RPE65 is one of the key markers of RPE cells and is responsible for light-independent conversion of all-*trans*-retinyl esters into 11-*cis*-retinol^58^. The data for CRALBP, a marker of hRPE maturation that is involved in retinol recycling^43^,^59^, were inconclusive with respect to whether this protein was significantly changed by addition of sericin. RPE65, however, was upregulated by sericin, as indicated by microarray profiling, RT-PCR and western blot. Thus, sericin affects at least some of the key markers of the visual cycle in cultured primary hRPE.

Viability analyses were performed to investigate whether the sericin-induced up-regulation of several inflammatory cytokines was accompanied with increased cell death. To reduce the effect of cell proliferation on cell density, the measurements of cell density were performed after 12 days of post-confluent culture with or without sericin or FBS. Sericin appeared to preserve cell density under serum-free conditions, and resulted in higher cell density than when adding either FBS or a combination of FBS and sericin to DMEM. Corroborating experiments with ETH-1 demonstrated that sericin promotes cell survival, which is in line with the down-stream prediction made by IPA. Our results are further supported by a study demonstrating that sericin protects against cell death following acute serum-deprivation^60^ and studies showing that FBS can be replaced by sericin in cryopreservation media without compromising viability^19^,^61^. The increased survival of hRPE upon stimulation with sericin may be related to up-regulation of *TNFAIP3* (*A20*), which inhibits TNF-induced apoptosis, or up-regulation of anti-oxidant genes, including the pigmentation-related gene *SOD2*. Downstream analysis by IPA also predicted a relationship between up-regulation of *TNFAIP3* and *SOD2* and increased cell viability and cell survival. In addition, a direct reactive oxygen species-scavenging effect of sericin has been reported elsewhere^62^. In RPE, inflammatory cytokines, such as IFN-γ and TNF-α, have been shown to induce *SOD2*, which promotes cell survival in the presence of oxidative stress^63^. Thus, even though sericin promoted augmented expression of the inflammatory NF-κB pathway, cell survival was increased, possibly by the concomitant up-regulation of anti-apoptotic and anti-oxidant genes.

In conclusion, sericin promotes pigmentation of cultured hRPE by activating the main NF-κB pathway. Sericin’s potential role in culture protocols for rapid differentiation of RPE cells derived from embryonic or induced pluripotent stem cells should be investigated.

## Methods

Normal primary hRPE and complete epithelial cell medium (EpiCM) were obtained from ScienCell Research Laboratories (San Diego, CA). Dulbecco’s modified eagle’s medium (high glucose, with pyruvate; hereafter named DMEM), Minimal Essential Medium (α-modification; MEM-α), heat-inactivated fetal bovine serum (FBS), N1 growth supplement, taurine, triiodo-thyronine, non-essential amino acids, glutamine-penicillin-streptomycin, hydrocortisone, propidium iodide (PI) and 4′,6-diamidino-2-phenylindole (DAPI) and 4-methyl-N1-(3-phenylpropyl)-1,2-benzenediamine (JSH-23) were obtained from Sigma Aldrich (St Louis, MO). Nunclon Δ surface plates, pipettes and other routine plastics came from VWR International (West Chester, PA). Mouse monoclonal anti-cellular retinaldehyde-binding protein (CRALBP; clone B2) was from Abcam (Cambridge, UK). Ethidium homodimer-1 (ETH-1) was from Invitrogen. Secondary Cy3-conjugated anti-mouse antibody was from Abcam. Ethidium homodimer 1 (EH-1) was obtained from Invitrogen (Carlsbad, CA). Soluene®-350 was purchased from PerkinElmer (Waltham, MA). Pierce BCA Protein Assay Kit was obtained from Bio-Rad (Hercules, CA).

### Cell Culture

Third passage hRPE were seeded (7000 cells/cm^2^) in complete EpiCM on Nunclon Δ surface plates and cultured under routine conditions of 95% air and 5% CO_2_ at 37 °C. A confluent layer was obtained after two days, at which time EpiCM was replaced with either: 1) DMEM with 1% FBS; 2) DMEM with 1% sericin; 3) DMEM without FBS or sericin; 4) MEM-α with 1% FBS; 5) MEM-α with 1% sericin; or 6) MEM-α without FBS or sericin. All culture media based on DMEM were supplied with 10.000 U penicillin and 10 mg streptymocin at a final concentration of 1%^43^. The MEM-α-based media were added taurine, triiodo-thyronine, non-essential amino acids, glutamine-penicillin-streptomycin, hydrocortisone, and N1 medium supplement, as described elsewhere^44^. The culture medium was changed every two to three days, and the hRPE were maintained in culture for a total of between nine to 14 days (two days in EpiCM and the remaining days in the DMEM or MEM-α based media).

### RNA Extraction and Microarray Hybridization

hRPE cells were cultured for 14 days and washed with PBS and lysed with QIAzol Lysis Reagent (N = 3). The lysate was transferred to a microcentrifuge vial. Total RNA was then purified according to the manufacturer’s protocol, and 100 ng of total RNA was processed with a GeneChip HT One-Cycle cDNA Synthesis Kit and a GeneChip HT IVT Labeling Kit (Affymetrix, Santa Clara, CA). Labeled and fragmented single stranded cDNAs were hybridized to the GeneChip Human Gene 1.0 ST Arrays (28,869 transcripts) (Affymetrix). Thereafter, the arrays were rinsed and stained using a FS-450 fluidics station (Affymetrix). Signal intensities were measured with a Hewlett Packard Gene Array Scanner 3000 7G (Hewlett Packard, Palo Alto, CA), and the scanned images were processed by the Affymetrix GeneChip Command Console (AGCC). The CEL files were imported into Partek Genomics Suite software (Partek, Inc. MO, USA). Robust microarray analysis (RMA) was applied for normalization. Gene transcripts with a maximal signal value less than 32 across all arrays were removed to filter for low and non-expressed genes, reducing the number of gene transcripts to 23190. Differentially expressed genes between groups were identified using one-way ANOVA analysis in Partek Genomics Suite Software. Clustering analysis was made using the same name module in a Partek Genomics Suite Software. Gene transcripts with maximal signal values of less than 5 were removed to filter for low and non-expressed genes, resulting in 14419 gene transcripts. For expression comparisons of different groups, profiles were compared using a 1-way ANOVA model. Data were presented as fold changes (FC) and *P*-values.

### Microarray Data Analysis

Upstream analysis, pathway analysis and downstream predictions were performed using Ingenuity Pathways Analysis (IPA) (www.ingenuity.com).

### Verification of Affymetrix Data by RT-PCR

The differential gene expression data were validated for selected transcripts using TaqMan® Gene Expression Assays and the Applied Biosystems®ViiA™ 7 Real –Time PCR system (Applied Biosystem, Life technologies, Carlsbad, CA) (Table 6). Affymetrix analysis identified *MAPRE1* and *POLR5H* as ideal for endogenous controls due to their stable expression across samples and groups. Briefly, 200 ng totalRNA were reverse transcribed using qScript™ cDNA Super Mix (Quanta Biosciences, Gaithersburg, MD) following the manufacturer’s instructions. After completion of cDNA synthesis 1/10 of the first strand reaction were used for PCR amplification. 9 *μ*l of cDNA (diluted in H_2_O), 1 *μ*l of selected primer/probes TaqMan^®^ Gene Expression Assays (Life Technologies) and 10 *μ*l TaqMan^®^ Universal Master Mix (Life Technologies) following the manufacturer’s instructions. Normalized ΔCt values were calculated by subtracting the average Ct of the two endogenous controls from the Ct of the reference gene. ΔΔCt values were then calculated by subtracting normalized ΔCt values of the group containing cells cultured in DMEM with 1% sericin from the control group, which included cells cultured in DMEM without sericin (N = 3). *P*-values were calculated using Student’s t-test in Microsoft Excel using delta Ct values. Normalized target gene expression levels (FC) were calculated using the formula: 2^(−∆∆Ct).

### Light Microscopy

Cell morphology and presence of pigment was assessed after 14 days of culture by light microscopy at 200× magnification (N = 8). Photomicrographs were captured using a Leica DM IL LED microscope and Canon EOS 5D mark II camera or with a Nikon Eclipse microscope with a DS-Qi1 black-and-white camera.

### Transmission Electron Microscopy

hRPE cells cultured for 12 days in DMEM with 1% sericin were processed for transmission electronmicroscopy (TEM) analysis as previously described^64^. In brief, ultrathin sections (60e70 nm thick) were cut on a Leica Ultracut Ultramicrotome (Leica, Wetzlar, Germany) and examined using a CM120 transmission electron microscope (Philips, Amsterdam, the Netherlands) (N = 4).

### Scanning Electron Microscopy

hRPE cells cultured on glass coverslips in DMEM with 1% sericin for 12 days were used for scanning electron microscopy (SEM) as described previously^65^. Glutaraldehyde-fixed samples (N = 3) were dehydrated in increasing ethanol concentrations and then dried according to the critical point method (Polaron E3100 Critical Point Drier, Polaron Equipment Ltd., Watford, UK) with CO_2_ as the transitional fluid. The specimens were attached to carbon stubs and coated with a 30-nm thick layer of platinum in a Polaron E5100 sputter coater before being photographed with an XL30 ESEM electron microscope (Philips, Amsterdam, The Netherlands).

### Melanin Measurement by Spectrophotometry

Intracellular melanin was quantified in hRPE cultured in DMEM with and without 1% sericin, JSH-23 and PMA. Seven-day cultures in 25 cm^2^ flasks were rinsed with PBS, and then re-suspended in 200 *μ*L of RIPA lysis buffer consisting of 25 mM Tris-HCL [pH 7.6], 150 mM NaCl, 1% NP-40, 1% sodium deoxycholate and 0.1% sodium dodecyl sulfate dissolved in H_2_O. Part of the cell lysates were then incubated with Pierce BCA protein assay kit for 30 minutes at 37 °C, and cooled to room temperature before measuring the absorbance at 562 nm using a microplate reader (VERSAmax, Molecular Devices, Sunnyvale, CA). Soluene®-350 was thereafter added to the remaining cell lysate (9:1). The cell lysate was subsequently incubated for 60 minutes at 80 °C, before being centrifuged for 10 minutes at 8600 g. Solubilized melanin was measured at 490 nm on the microplate reader, and the concentration was adjusted by multiplication with protein levels (N = 5).

### Quantitative Immunofluorescence

Following 14 days of culture the cells were fixed in 100% methanol for 15 minutes and then washed three times with fresh PBS (N = 4–8). Fixed cells were incubated for 45 minutes at room temperature in a blocking buffer consisting of 10% goat serum, 1% BSA, 0.1% Triton X-100, 0.05% Tween-20, 0.05% sodium azide in PBS. Cells were then incubated overnight at 4 °C with the following antibody diluted in blocking buffer: anti-CRALBP (1:100), which targets a functional protein in the visual cycle and a marker for differentiated hRPE. The Cy3-conjugated secondary antibody, diluted in 0.2% PBST with 1% BSA, were incubated for one hour at room temperature. Negative control consisted of replacing the primary antibody with PBS. The cultures were thereafter rinsed three times in PBS and incubated with 1*μ*g/mL DAPI in PBS to stain cell nuclei before a final wash with PBS.

Photomicrographs of the cultures were captured at 200× magnification using a Nikon Eclipse Ti fluorescence microscope with a DS-Qi1 black-and-white camera. The exposure length and gain was maintained at a constant level for all samples, and the fluorescence intensities of the Cy3 fluorochromes, which were conjugated to the secondary antibodies, were within the dynamic range of the camera.

Phenotype was quantified using ImageJ (National Institutes of Health, Bethesda, MD) as described elsewhere^66^,^67^, with some modifications. In brief, mean fluorescence per cell was measured by enlarging regions of interest (ROI) created around the DAPI-stained nuclei to enclose the CRALBP-expressing cytosol. By using this method, we were able to normalize for differences in cell density in each photomicrograph.

### Quantification of Cell Density

After 14 days in culture, the hRPE (N = 4–8) were fixed with methanol, as described above. The cultures were then rinsed three times in PBS and incubated with 1 *μ*g/mL DAPI in PBS to stain cell nuclei before a final wash with PBS. Photomicrographs of the cultures were captured at 200× magnification with identical exposure length and gain. ImageJ was used to convert 16-bit images to binary images, as reported elsewhere^66^. The “Analyze particles” function in ImageJ was then used to automatically count cell nuclei per image.

### Quantification of Cell Death

The amount of dead cells in the cultures after 14 days was quantified by incubating the samples with EH-1 for 30 minutes at 37 °C (N = 8). Ethidium homodimer-1 stains nuclei of dead cells red and its fluorescence was quantified by the microplate fluorometer with the excitation/emission filter pair 530/620. Background fluorescence, measured in wells incubated with EH-1-reagent, but without cells, was subtracted from all values before calculating mean fluorescence for the groups.

### Western Blot

Protein levels were measured by Western blot analysis of primary hRPE cultured for 12 days. In brief, cells were lysed in RIPA-buffer and proteins analyzed by SDS-PAGE followed by electroblotting onto PVDF membranes.

The membranes were blocked with 5% bovine serum albumin/TBS at ambient temperature and incubated over-night at 4 °C with rabbit polyclonal anti-CRALBP (1:1500), mouse monoclonal anti-RPE65 (1:5000) or mouse monoclonal anti-NF-κB p100/p52 (1:4000). Samples were then incubated for one hour at ambient temperature with HRP-conjugated goat anti-rabbit (1:10.000) or goat anti-mouse (1:10.000) secondary antibodies. Immunoreactive bands were detected using enhanced chemiluminescence (ECL) substrate (Pierce Chemical Co, Rockford, USA), and visualized using the ImageQuant LAS4000 system (GE Healthcare, USA). To confirm the equality of protein lysates loading, membranes were subjected to mild stripping as described (http://www.abcam.com/ps/pdf/protocols/stripping%20for%20reprobing.pdf), and re-probed using a mouse anti-actin monoclonal antibody (1:3000), then processed as above. Intensity of the protein bands were quantified by ImageJ.

### ELISA

Level of phospho-NF-κB p65 was measured by enzyme-linked immunosorbent assay (ELISA) in primary hRPE cultured for 12 days according to the manufacturers instructions (RayBio® Phospho-NF-κB p65 (S536) and Total NF-κB p65 ELISA kit). Briefly, samples were incubated with anti-phospho-NF-κB p65 for 2.5 hours at ambient temperature, washed, incubated with primary antibody for 1 hour, washed and then incubated with HRP-conjugated anti-rabbit IgG. Following washing, the addition of TMB substrate solution and Stop Solution, the color changes were measured at 450 nm.

### Statistical Analysis

One-way ANOVA with Tukey’s (equal variances assumed) or Dunnett’s T3 (equal variances not assumed) post hoc pair-wise comparisons were used to compare three or more groups, while Student’s t-test was used to compare two groups (SPSS ver. 21.0). Data were expressed as mean ± standard deviation, and values were considered significant if *P* < 0.05.

## Additional Information

**How to cite this article**: Eidet, J. R. *et al*. The Silk-protein Sericin Induces Rapid Melanization of Cultured Primary Human Retinal Pigment Epithelial Cells by Activating the NF-κB Pathway. *Sci. Rep.* **6**, 22671; doi: 10.1038/srep22671 (2016).

## Figures and Tables

**Table 1:** Differentially Expressed Genes in the NFκB-pathway in hRPE Cultured in DMEM with Sericin.

**Gene Symbol**	**Gene Name**	**Affymetrix ID**	FC	***P*****-value**
TNFAIP3	Tumor necrosis factor, alpha-induced protein 3	17012946	10.4	8.7E-05
MAP3K8	Mitogen-activated protein kinase kinase kinase 8	16703642	2.5	1.1E-02
NFKB2	Nuclear factor of kappa light polypeptide gene enhancer in B-cells 2 (p49/p100)	16708623	2.1	3.3E-05
TNFSF13B	Tumor necrosis factor (ligand) superfamily, member 13b	16776339	2.1	1.1E-03
NFKB1	Nuclear factor of kappa light polypeptide gene enhancer in B-cells 1	16969300	2.0	2.3E-04
TNFAIP3	Tumor necrosis factor, alpha-induced protein 3	17012946	1.8	8.7E-05
TNIP1	TNFAIP3 interacting protein 1	17001763	1.6	3.5E-04
RELB	V-rel avian reticuloendotheliosis viral oncogene homolog B	16863168	1.6	9.0E-04
EGF	Epidermal growth factor	16969729	1.4	1.1E-02
MAP3K1	Mitogen-activated protein kinase kinase kinase 1, E3 ubiquitin protein ligase	16984945	1.2	1.5E-02
TGFA	Transforming growth factor, alpha	16898788	1.1	4.5E-02

hRPE = human retinal pigment epithelial cells; DMEM = Dulbecco’s modified eagle medium; FC = fold change.

**Table 2:** Top Ten Up- and Down-regulated Genes in hRPE Cultured in DMEM with Sericin.

**Gene Symbol**	**Gene Name**	**Affymetrix ID**	FC	***P*****-value**
CXCL10	Chemokine (C-X-C motif) ligand 10	16977052	55.5	4.2E-06
C3	Complement component 3	16867784	34.7	1.2E-06
CCL2	Chemokine (C-C motif) ligand 2	16833204	21.6	9.3E-07
IL6	Interleukin 6	17044177	15.6	8.8E-06
TNFAIP3	Tumor necrosis factor, alpha-induced protein 3	17012946	10.4	8.7E-05
ICAM1	Intercellular adhesion molecule 1	16858137	9.1	2.0E-06
PTX3	Pentraxin 3, long	16947357	8.0	1.4E-04
ADA	Adenosine deaminase	16919466	7.7	2.6E-06
EDNRB	Endothelin receptor type B	16779958	7.5	1.6E-05
CNTNAP1	Contactin associated protein 1	16834409	7.3	5.3E-06
CLGN	Calmegin	16980051	−5.3	4.0E-06
ADAM28	ADAM metallopeptidase domain 28	17066921	−5.5	2.8E-05
LUZP2	Leucine zipper protein 2	16722987	−5.6	8.7E-05
VCAN	Versican	16986913	−6.4	2.6E-05
MFAP4	Microfibrillar-associated protein 4	16842266	−6.6	3.9E-06
LRRC15	Leucine rich repeat containing 15	16962911	−6.9	3.2E-04
ST8SIA4	ST8 alpha-N-acetyl-neuraminide alpha-2,8-sialyltransferase 4	16998532	−6.9	7.3E-06
COL14A1	Collagen, type XIV, alpha 1	17072162	−9.2	4.5E-07
SMOC2	SPARC related modular calcium binding 2	17014798	−9.8	2.8E-08
GRIK3	Glutamate receptor, ionotropic, kainate 3	16685330	−16.4	5.3E-06

hRPE = human retinal pigment epithelial cells; DMEM = Dulbecco’s modified eagle medium; FC = fold change.

**Table 3:** Differentially Expressed Pigmentation-associated Genes in hRPE Cultured in DMEM with Sericin.

**Gene Symbol**	**Gene Name**	**Affymetrix ID**	**FC**	***P*****-value**
IL6	Interleukin 6	17044177	15.6	8.8E-06
EDNRB	Endothelin receptor type B	16779958	7.5	1.6E-05
SOD2	Superoxide dismutase 2, mitochondrial	17025267	4.6	1.6E-05
C10orf11	Chromosome 10 open reading frame 11	16706350	3.1	1.1E-05
IRF1	Interferon regulatory factor 1	16999776	3.0	1.0E-03
PROM1	Prominin 1	16974534	2.9	2.6E-05
INSIG1	Insulin induced gene 1	17053892	2.9	1.2E-04
SLC24A5	Solute carrier family 24, member 5	16800764	2.2	1.8E-04
SOD3	Superoxide dismutase 3, extracellular	16965519	2.1	4.1E-04
TGM2TGF2	Transglutaminase 2	16919158	2.0	6.9E-04
HELLS	Helicase, lymphoid-specific	16707695	−2.0	3.4E-03

hRPE = human retinal pigment epithelial cells; DMEM = Dulbecco’s modified eagle medium; FC = fold change.

**Table 4:** Differentially Expressed Visual Cycle-associated Genes in hRPE Cultured in DMEM with Sericin.

**Gene Symbol**	**Gene Name**	**Affymetrix ID**	**FC**	***P*****-value**
RPE65	Retinal pigment epithelium-specific protein 65kDa	16688370	2.8	2.7E-05
RDH10	Retinol dehydrogenase 10 (all-trans)	17070013	1.9	1.7E-05
RDH11	Retinol dehydrogenase 11 (all-trans/9-cis/11-cis)	16794064	1.7	3.7E-04
RLBP1 (CRALBP)	Retinaldehyde binding protein 1	16813062	1.4	8.0E-04

hRPE = human retinal pigment epithelial cells; DMEM = Dulbecco’s modified eagle medium; FC = fold change.

**Table 5:** Verification of Affymetrix data by RT PCR.

**Gene symbol**	**Gene Name**	**RT PCR**		**Affymetrix**	
		**FC**	***P*****-value**	**FC**	***P*****-value**
NFKBIA	Nuclear factor of kappa light polypeptide gene enhancer in B-cells inhibitor, alpha	12.9	4.0E-06	6.9	2.0E-03
RPE65	Retinal pigment epithelium-specific protein 65kDa	4.3	1.8E-07	2.8	2.7E-05
CSF1	Colony stimulating factor 1 (macrophage)	6.8	1.8E-04	2.8	1.0E-02
NFKBIZ	Nuclear factor of kappa light polypeptide gene enhancer in B-cells inhibitor, zeta	5.1	2.0E-03	2.6	2.0E-02
RGR	Retinal G protein coupled receptor	3.6	5.4E-05	2.3	2.0E-03

The table shows average fold change (increase) in mRNA levels upon incubation of human retinal pigment epithelial cells with sericin for 12 days as compared to controls. Data are average from triplicates with MAPRE1 and POLR5H as endogenous controls. RT PCR = real time polymerace chain reaction; FC = fold change.

**Table 6:** Taqman Assays.

**Gene symbol**	**Assay ID**
RPE65	Hs01071462_m1
RGR	Hs00173619_m1
NFKBIA	Hs00355671_g1
NFKBIZ	Hs00230071_m1
CSF1	Hs00174164_m1
POLR3H	Hs00978014_m1
MAPRE1	Hs01121102_g1
